# Clinical Report for the Academic Years 1835-7

**Published:** 1840-01

**Authors:** 


*Rendiconto Clinico per gli Anni Accademici*, 1835-36, *e* 1836-37. Di CARLO GIACINTO SACHERO, Professore di Clinica Medica nella regia Università di Torino.—*Torino*, 1838. 8vo, pp. 356.

*Clinical Report for the Academic Years* 1835-6, *and* 1836-7. By C. G. SACHERO, Professor of Clinical Medicine in the Royal University of Turin.

FOURTEEN beds, our author tells us, are allotted in the general hospital, for the purpose of clinical instruction; "a small number, it may seem at first sight, to those who like to wander over many objects, without fixing their view on one; and who, miscalculating their own powers, believe that they have lynx's eyes." But the professor satisfies himself with an aphorism from the learned Stoll, "multa non multum videre volunt, qui maximum ægrorum numerum videndi prurigine tentantur."

The diseases of those received into the clinical wards are usually severe. The first four months of the academic year are devoted to the males' wards, the last four to the females'; and, in the course of these eight months, from 130 to 160 cases are presented for observation. A certain number of pupils are attached to each bed, whose office it is to attend the patients by turns, and, on the admission of each, to obtain information concerning his or her history, symptoms, and condition, from which they have to make accurate reports to the professor, and to form their own diagnosis. The professor himself next examines the patient, and confirms or annuls the pupil's opinion. The latter has then to keep a daily record, which constantly lies at the bedside, to be consulted by the other pupils, and which, at the conclusion of the case, is reduced into a proper form, with the minutes of the public dissection in those which terminate fatally.

The volume before us is composed of cases thus written, and of the professor's remarks upon them, and upon pathology in general; and though none of them are of remarkable interest, the work affords a fair idea of the present condition of both practical and scholastic medicine in this part of Italy. The cases are arranged according to an appended nosological table, in which the chief divisions are founded on locality. We have first, "Diseases of the vascular system," including periodical and inflammatory fevers. In most of these cases, "some rapid bleedings, gentle purgatives, soothing drinks or diaphoretics, abstinence and rest, were sufficient to overcome the irritation, and to prevent their passing into *angioitis*." Here we come at once to the prominent feature of the work. This angioitis is a disease, to the name of which most Italian physicians are much devoted, and they class under it many different affections, in which the symptoms during life are by no means positively indicative of any inflammation of the vessels, and in which, after death, the only evidence of such a disease is redness of the interior of the aorta and its branches. The following case, for example, is given as one of *angioitis acuta:*

"A servant girl, aged thirteen, of sanguine temperament, rather fond of wine (un po' amica del vino), and often exposed to atmospheric changes, became ill on the last day of May, and came into the hospital on the 1st of June. On her admission she was bled to ten ounces, and in the evening she presented the following symptoms: frequent palpitation of the heart, very acute pulsating pain of the head, increased by motion, face flushed, eyes glistening, skin very hot, tongue red at its edges, and mucous in the middle, respiration anxious, pulse *cardiaco-cephalic*, vibrating, metallic, pretty frequent. Diagnosis: *Angioitis.* Another bleeding was ordered in the course of the evening, and cooling drinks. On the three following days the symptoms varied but little, and she was bled five times more, and took digitalis. In the three next days the violence of the fever abated, but the pain of the head continued, and required two leeches (!) to be applied to the mastoid region. An exasperation of the symptoms occurred on the evening of the 10th, for which she was bled again on the following morning. On the sixteenth day of her disease she had sweat; the symptoms then gradually diminished, and she left the hospital on the twenty-ninth." (p. 63-4.)

There is nothing in this history to indicate that the patient had more than a tolerably severe attack of inflammatory fever, unless, indeed, the ability of a girl of thirteen to bear eight considerable abstractions of blood in ten days, be supposed to prove some more serious disease. Yet this seems a good example of that which is commonly called angioitis, and for which (its importance being much exaggerated by long arguments) it is thought necessary to employ the most severe treatment.

But angioitis exists in diseases the very antipodes of inflammatory fevers, as in the next case, which is one of *angioitis lenta*, or *arteriasis:*

"A countrywoman, aged twenty-two, born of cachectic parents, and with a pallid countenance, had for more than a year suffered from suppressed menstruation, after having bathed. She then began to have dyspnæa, on going up hill, palpitation of the heart, weight in the head and loins, loss of appetite, and other similar symptoms, which increased till she could no longer work. When she came into the hospital she had a pulsating pain in the forehead, and a pale sallow face; her tongue was red at the margins; she had thirst, nausea, weight in the epigastrium, and in the precordial region; a feeling of anxiety and suffocation, and throbbing of the carotids; the action of the heart was increased in quickness and in impulse, the pulse *cardiac*, slightly *stomachal*, tense, and frequent, the skin hot. Diagnosis: *Angioitis lenta*, with slight gastritis. She was bled at once (the amount is not stated), and again on each of the three following days; digitalis was also given, and twenty-seven leeches applied to the epigastrium. After these means, the symptoms diminished in severity; but on the twelfth day after her admission they again returned, and there seemed to be a *conatus hemorrhagicus*, the pulse being cardiaco-uterine: leeches were applied to the groins, and castor-oil was given. Next day she was better: pediluvia of mustard, and pills with iron-filings, digitalis and hyoscyamus were ordered, to restore the catamenia; they, and nutritious food, succeeded in that intention, and the patient was cured." (pp. 77-8.)

This was, assuredly, only a common case of the effects of suppressed menstruation, which the means at last employed would probably have cured at the first. But between chlorosis and inflammatory fever, nearly every disease accompanied by much palpitation of the heart, and throbbing of the arteries, seems to be called and severely treated as angioitis. It forms, too, an important ingredient in many other diseases. Thus, in a number of the Transactions of the Medico-Chirurgical Society of Bologna, which we lately read, a case was detailed by Dr. Golinelli, in which a woman had a portion of intestine completely strangulated by a band of false membrane. She died with all the symptoms of strangulated hernia, and on examining the body, twenty-four hours after death, in addition to acute suppurative peritonitis from the intestinal obstruction, the lining of the heart and aorta was found reddened, especially where some coagula of blood lay in it. Nearly the whole of the rest of the very long paper was occupied by a most learned discourse on the question whether the patient had died of the strangulated intestine, or of arteritis; and the author summed up rather in favour of the latter!

Now it is evident that the symptoms detailed in these cases are quite insufficient to establish the existence of an inflammation of the arteries; and the only other fact on which it is assumed is, that their interior is sometimes found red after death. The inadequacy of the evidence which this affords has often been remarked on; but, without quoting authorities on each side, the resultant of whose opposite opinions might still be uncertain, we would observe, that if it is considered how acute any inflammation must be, in a slightly vascular tissue, to make it red after death,—how, in many tissues, of which the inflammatory redness can be seen during life, it disappears when life ceases, as in the skin, conjunctiva, &c.—and how improbable it is that a tissue so little vascular as the middle coat of arteries, possessing as it does even fewer vessels than the tendons, should ever, by any extent of inflammation, become red—one might expect, that arteritis would not have redness at all for a sign, or at least would not have it without other coincident results of most acute inflammation, as effusion of lymph, thickening, &c. And when to these one adds those facts which have been more generally observed, that the arteries, and many other tissues, will assume an internal redness long after death, when they are exposed to blood, and that this same redness is often found only where blood is in contact with the wall of the artery, and very rarely unless several hours have elapsed after death; while sometimes, on the other hand, no very evident redness exists in cases in which the other more determined results of inflammation are present;—the conclusion seems certain, that the redness of the arterial lining is no evidence that the artery has been inflamed. The propriety or safety, therefore, of the copious bleedings and severe antiphlogistic treatment with which palpitations of the heart and throbbings of the arteries are met by the Italian physicians, may well be doubted.

We have next three cases of puerperal phlebitis, of which two recovered by copious and repeated general and local bleedings; in one, in which the symptoms of inflamed varicose veins of the leg were by no means very severe, the patient lost a pound of blood seven times in four days, and had thirty-six leeches applied!

The next section is on diseases of the heart, in which there is nothing remarkable, except a boldness of diagnosis of pericarditis in every case, without a complete assurance of its existence by the stethoscope, and a boldness of antiphlogistic treatment fully proportioned to the phlogistic doctrine.

The same ideas are prominent in the section on cerebral affections, and to all those of the spinal cord we have still the same formidable termination,—*itis.* Among the latter, there is a case of convulsive hysteria, under the name of *myelitis membranosa lenta cum orgasmo uterino*, and two cases of sciatica and lumbago, which are raised to the titles of *myelitis membranosa lumbo-sacra*, and *spinitis sacra.* In all, very liberal abstractions of blood were the chief or only means employed.

In short, throughout the whole work, we look in vain for anything but inflammations, and the severe though simple treatment which, if they existed, they would scarcely need. But these are faults, not of the professor himself, who seems an industrious and judicious teacher, a bold practitioner, and at the same time a well-read scholar, but of the time and country in which he lives. The doctrine of inflammation in everything, is almost universally prevalent in Italy, and a large majority of the physicians add to their faith in it, a most vigorous practice against it.

The result of this general adoption of an unproved theory is, we need not say, most mischievous in every respect; but in none—not even in its exclusion of useful remedies, because completing the theoretical circle, they are not thought anti-inflammatory—in none, more than in the obstacle which it presents to all open and unprejudiced enquiry. To the one wide-expanding and all-explaining word, every observation is made to bend, and by it the whole current of enquiry is perverted or restrained.

The Italian physicians appear still to be guided by that spirit of hypothesis which, from the historic origin of medicine to the middle of the last century, held almost universal sway in every medical school, and on which, indeed, the very existence of the schools, and the reputation of the schoolmen depended. They are still striving after some expression even more extensive than inflammation, that may conceal from themselves their own ignorance of the real truths of nature. They have an Archæus in full power, if not in name—excitement and excitability—sthenia, asthenia, and hypersthenia—and all the portentous titles from which, ingeniously woven into an hypothesis, (strangely flimsy, considering the weight of its materials,) they may reason downwards to the presumed explanation of some simple facts.

Unless it be that there is the same deficiency of inventive genius among the Italians now as in their better days, it is difficult to say why this spirit of the schools should yet find a stronghold among them, after it has been driven from its once firm seat in England, France, and Germany. It is the more remarkable, because to Italy we owe the very founders of that school of physiology whose pupils were destined to supplant alike both empirics and theorists. From Italy the spirit of scientific observation spread over the rest of civilized Europe, and though checked for a time, by the popularity of the doctrinal teaching of Germany, has obtained, at last, almost an entire control. In its birthplace alone has it lost any of its just domain, and been deposed by the plausible false wisdom of "the schools," which it has elsewhere conquered.

Could they but be induced fully to appreciate the value of observed or demonstrated truth, and clearly to see its superiority over any hypothesis, however wide, or seemingly sufficient, the physicians of Italy might at once occupy the highest rank in Europe, for then they could make that an important auxiliary, which is now too often a burden—their intimate acquaintance with the literature, not only of medical science, but of sound and refined philosophy—a kind of learning with which all their works run over, and in which their brethren of other nations cannot for a moment compete with them.

